# Weighted Similarity and Core-User-Core-Item Based Recommendations

**DOI:** 10.3390/e24050609

**Published:** 2022-04-27

**Authors:** Zhuangzhuang Zhang, Yunquan Dong

**Affiliations:** 1 School of Electronic and Information Engineering, Nanjing University of Information Science and Technology, Nanjing 210044, China; zhuang.zhang@nuist.edu.cn; 2 National Mobile Communications Research Laboratory, Southeast University, Nanjing 210096, China

**Keywords:** weighted similarity, recommendation, core users, core items

## Abstract

In traditional recommendation algorithms, the users and/or the items with the same rating scores are equally treated. In real world, however, a user may prefer some items to other items and some users are more loyal to a certain item than other users. In this paper, therefore, we propose a *weighted similarity* measure by exploiting the difference in user-item relationships. In particular, we refer to the most important item of a user as his *core item* and the most important user of an item as its *core user*. We also propose a Core-User-Item Solver (CUIS) to calculate the core users and core items of the system, as well as the weighting coefficients for each user and each item. We prove that the CUIS algorithm converges to the optimal solution efficiently. Based on the weighted similarity measure and the obtained results by CUIS, we also propose three effective recommenders. Through experiments based on real-world data sets, we show that the proposed recommenders outperform corresponding traditional-similarity based recommenders, verify that the proposed weighted similarity can improve the accuracy of the similarity, and then improve the recommendation performance.

## 1. Introduction

With the rapid development in Internet technology, there has been an explosive growth in the amount of information on the Internet. For each single user, therefore, it is quite difficult to find the information they are really interested in. To this end, many efforts have been made to help users to identify those information they really need, such as the information retrieval technology and the information filtering method. The most popular tool for information retrieval is the search engine. Although they are widely available to users, however, search engines fail to satisfy the individual demands and preferences of each user. Therefore, various information filtering based recommendation systems, which are designed to meet users’ special needs, have been widely investigated [1].

As one of the most effective recommendation methods, Collaborative Filtering (CF) based recommenders make recommendations by learning user-item preference patterns from a large amount of historical data [2,3]. In particular, CF can be implemented by the memory-based (neighborhood-based) methods or the model-based methods. In the neighborhood-based methods, we first find some of the most similar users (friends) for a user, and then recommend some popular items among these friends to the user. We can also find out those items similar to the preferable items of a user and recommend these items to him. Since the performance of recommendations is largely determined by how accurately similar users (or items) are found, the metric measuring the similarity among users (items) is crucial. Among the widely used similarity measures such as the Cosine similarity, the Pearson correlation coefficient, the Euclidean distance inverse, and the Jaccard similarity coefficient [4], we note that the users and items are *treated equally*. That is, users (items) with the same rating scores are used and processed with no difference. There have been many works studying weighted similarity based schemes. However, most of the current weighting schemes [5] are humanly devised and computed by predefined functions. Thus, it is unclear about what the optimization objectives those weighting schemes are trying to achieve and whether they are reasonable. For example, the automatic weighting schemes proposed in [6,7,8] assumed that the weighting coefficients of items are same for all users. However, the weighting coefficients of items for different users should be different from each other, since the tastes of the users are different. In addition, the weighting coefficients of the users to items were rarely considered in the previous literatures. In everyday life, however, we often observe that a certain user likes some items much more than others, and some users are more fanatical to an item than others.

**Example** **1.** We show a rating matrix with five movies and five users in Table 1. For each movie, we summarize the rating scores made by the users as a row of the matrix. For each user, his rating scores are all presented in the corresponding column. If a user has not watched and rated a certain movie, then the corresponding element of the matrix is denoted by *.

*First, it is clear that a user likes a set of movies differently if he has rated them differently. For example, Joe likes Star Wars more than Frozen. Second, it is readily seen that Bob’s favorite movie is Frozen and the most loyal user of Frozen is Bob. In this sense, Bob is the most recognizable user for Frozen and reflects the main characteristics of users voting for it. Third, we observe that Ali rated three of the movies with full scores. Thus, it is not clear which movie he likes best. Likewise, three of the users rated Star Wars with full scores and it is difficult to tell which user likes the movie the most. This is mainly because the precision of both the pre-defined system scores and the rating capability of the users are very limited. That is, the rating scores are limited to integers between 1 and 5 while the users can only evaluate their preferences approximately and randomly.*

*For example, we assume that Ali is an adult college student. Ali likes Star Wars due to the spectacle of the film and likes WALL·E since he is touched by the delicate emotional story. However, his true favorite is Inception since he is a sci-fi fan. In addition, the elements like passion and imagination are crucial to him. Although we cannot observe his preference to sci-fi and how he likes the three movies differently from his 3 full scores, we do can obtain more information from the movie he does not like. Based on the fact that he does not like the relatively childish ‘Frozen’, it would be reasonable to deduce that he likes Inception more than the other two movies.*

*Therefore, it is important to investigate the differences of the users’ preferences for movies and the importance of the movies to users in terms of two sets of optimized weighting coefficients. By defining new similarity measures based on these weighting coefficients, we can also evaluate the similarity among users and among movies more accurately.*

In this paper, therefore, we further exploit the relations between the users and between the items by considering the difference in the degrees of preference. In particular, we propose a new metric of similarity, which is referred to as the *weighted similarity*, to quantify the similarity between users and between items. Moreover, we propose a *Core-User-and-Item-Solver (CUIS)* to find the core (most favorable) items of each user, the core (most fanatical) user of each item, and the weighting coefficients for all the users and items. Based on these information, we further propose three recommenders and verify their effectiveness via real-world data sets.

The contributions of the paper are summarized as follows.

- We propose a *weighted similarity* measure to quantify the similarity between users and between items, and to explore the difference in the degrees of importance of the items (to users), as well as of the users (to items).
- We propose a CUIS algorithm to find the weighting coefficients of the items and users, the core items of users, and the core users of items.
- Based on CUIS and the weighted similarity, we propose three effective recommendation methods. Through experiments based on real-world data sets, we also verify that the proposed weighted similarity measure can improve the accuracy of the similarity and improve the recommendation performance.

Table 2 provides a check list of the frequently used notions throughout the paper.

### 1.1. Related Work

The recommendation algorithm is the core of the recommendation systems, and its performance determines whether the final recommendations are accurate or not. In general, recommendation algorithms fall into four categories: content-based filtering (CBF), collaborative filtering (CF), hybrid methods and deep learning methods. Content-based filtering techniques try to learn a feature-based representation of the content of recommendable items [9]. Specifically, these approaches aim to recommend items which are similar to that the user liked in past or is looking at present [10]. Albatayneh et al. [11] presented a content-based preference learning model called “Discriminate2Rec”, which discriminates between items’ attributes based on their influence on user temporal preferences. The advantages of content-based recommender systems include simplicity, transparency, independence, and immune to the cold start problem. Content-based recommendation algorithms also have many drawbacks, including serendipity, heterogeneity of information, and low accuracy. The CF approaches include memory-based (neighborhood-based) CF and model-based CF. Memory-based CF can be classified into item-based CF and user-based CF, in which item-based CF methods [12] recommend items similar to those items loved by the same user, and user-based CF methods [13] suggest items to target users that their neighbors like. Model-based CF uses data mining techniques or machine learning algorithms to learn a model, which is then used to make rating predictions [14]. Compared with CBF methods, memory-based CF has some merits, including sharing the experience of others, avoiding the incompleteness and imprecision of content analysis, and providing serendipitous recommendations. Moreover, memory-based CF methods are also easy to implement, relatively simple, and are able to present intuitive explanations of the recommendations. The demerits of memory-based CF methods include sparsity, scalability, and cold start problems. Compared with memory-based CF, which only uses one dimension to express data (either users or items), a model-based CF is constructed from two dimensions of users and items. Therefore, model-based methods have stronger expression ability in describing all aspects of data and output better predictions. Model-based CF makes use of all scoring information, so it is more effective for the overall evaluation. Neighborhood-based CF only considers a small part of the main key ratings, so it is more effective for evaluating localization relationships. These methods when used individually have many complementary merits and demerits. This fact has provided a stimulus for research in hybrid recommender systems, which combine various techniques to improve performance. Various hybrid methods have been proposed, including the hybridization techniques (weighted, mixed, switching, feature combination, feature augmentation, and meta-level) [15]. Hybrid methods can deal with the problems of cold start and sparse data, improve the robustness and scalability of the system, and improve the accuracy of recommendation. However, the disadvantages are also obvious. That is, the integration of multiple algorithms inevitably increases the overall algorithm complexity and occupies a lot of memory and computing resources. Compared with the above mentioned traditional methods, deep learning methods automatically learns features from multi-source heterogeneous data and the deep interactive relationship between learning features. Deep learning models commonly used in the recommendation system include: multi-layer perceptron [16], autoencoder [17], convolutional neural networks [18], recurrent neural networks [19], generative adversarial networks [20,21], etc. Although deep learning recommendation methods can generate a very good recommendation effect, it suffers from interpretability difficulties and extensive hyper-parameters adjustment.

Despite the long history of the neighborhood based CF methods, they are still widely used in both research and industry (such as in research management areas [22] and Amazon.com (accessed at 22 April 2022) [23]) because of its advantages of easy implementation, good interpretability and intuitive simplicity. For this kind of recommendation algorithms, one of the key problems is how to measure the similarity among users and items [24]. Therefore, improving the accuracy of similarity plays a decisive role in improving the recommendation performance.

There have been many works studying the similarity among users and items. Researchers first used the distance between two vectors to calculate the similarity of items or users. Among them, the Euclidean distance [25] is the most commonly used distance measure, which is measured by the absolute distance of each point in the two multi-dimensional vectors. Euclidean distance needs to ensure that the indicators of each dimension are at the same scale level. For two vectors with different scales, it is necessary to standardize the data first and then use Euclidean distance to derive Mahalanobis distance [26]. These distance measurement methods are more used in the research that needs to reflect the difference from the individual value size of each dimension. Therefore, researchers began to use correlation based methods to measure the similarity in neighborhood-based CF methods. Singh et al. [27] used the cosine similarity to measure the similarity between two vectors. However, this method did not consider the difference between the mean and variance of each item’s ratings. The authors used Pearson correlation coefficients (PCC) [28] to eliminate this effect, which centralized item ratings by subtracting the average of the elements from all dimensions in the two vectors. Musa et al. [29] also used adjusted cosine similarity to improve similarity accuracy by considering the average value of centralized user ratings, and found that in the case of item-based CF recommendations, this similarity performs better than PCC. In addition, another correlation-based similarity is Spearman rank correlation coefficients (SRCC) [30]. PCC uses the score value, while SRCC uses the hierarchical order of these scores, that is, the similarity value is independent of the specific value of each element of the two vectors, but only related to the size relationship between its values. Although SRCC avoids the problem that the score must be standardized, when there are many score values, a large number of grade values will be generated. SRCC needs to calculate these grades, so it will incur more calculation cost. Some Jaccard similarity and its variants [31,32] are mainly used in binary implicit feedback recommendation systems. In Ref. [7], the authors investigated the performance of several similarity measures, including PCC, SRCC, the vector “cosine” measure, the entropy-based uncertainty measure, and the mean-squared difference. It is observed that PCC and SRCC can exploit the correlation among users and items better than other measures. Howe et al. [33] found that the calculation accuracy of similarity is also related to the data itself through experimental analysis. The literature [4] summarized common similarity based on neighborhood recommendation and analysed their advantages and disadvantages. However, all of these similarity measures treat the items and users with the same scores equally. Thus, some weighting schemes were investigated, such as inverse user frequency based method [6], entropy and mutual information based method [5], the variance weighting scheme [7] and the automatic weighting scheme [8]. In Ref. [6], the authors proposed an inverse user frequency (IUF) for weighting different items. More specifically, the IUF weight for an item *i* is defined as $w_{i}^{\mathrm{IUF}}=\log\left(N/N_{i}\right)$ where *N* is the number of training users and $N_{i}$ is the number of training users that have rated item *i*. The IUF weights favor the items that have been rated by a few users. However, the items rated by fewer users may not necessarily be useful in telling users of different tastes. The results in [5] showed that the IUF weighting has degraded the performance of the PCC method. The authors [7] considered a variance based weighting scheme. It is based on the intuition that an item with a larger rating variance is more valuable in discerning the true interests of the users than an item with a smaller variance. Specifically, the weight for item *i* is computed as $w_{i}^{\mathrm{VW}}=\frac{{var}_{i}-{var}_{\min}}{{var}_{\max}}$ where ${var}_{i}=\frac{\sum_{u=1}^{N}{\left(r_{ui}-\bar{r_{i}}\right)}^{2}}{N-1}$, and ${var}_{\min}$ and ${var}_{\max}$ represent the minimum and maximum variances over all items. However, this is not necessarily true because a large variance in the ratings of an item can also arise from the difficulty in rating such an item by many users. As shown in [7], the variance weighting scheme leaded to slightly worse performance than those schemes without weighting. In [8], the authors proposed a probabilistic model for measuring the similarity between different users that can incorporate weights for different items and formulated the problem of finding appropriate weights for each item into an optimization problem to solve. However, the same set of weight coefficients (for items) are used for all users, which may diverge from the true tastes of the users. It is noted that, these weighting schemes only considered a set of weights of items for the system, ignoring the differences of different users’ preferences for items, thus failed to find the personalized item-weighting coefficients for different users. In addition, these schemes did not consider the users’ weights (to items) to design the weighted similarity. In this paper, therefore, we further improve the similarity between the users and between the items by considering the difference in the degree of preference of different users for the items and the importance of different items to the users.

### 1.2. Organizations

The rest of the paper is organized as follows. In Section 2, we present the definition of the weighted similarity between users and between items, and illustrate how the CUIS algorithm works. In this section, we also prove the optimality and the convergence of CUIS. In Section 3, we propose three recommenders by combining the traditional recommenders and the obtained results on core users/items and weighted coefficients. We verify the effectiveness of CUIS and proposed recommenders in Section 4 and conclude the paper in Section 5.

## 2. The CUIS Algorithm

### 2.1. Weighting Coefficients and Weighted Similarity

**Figure 1:**
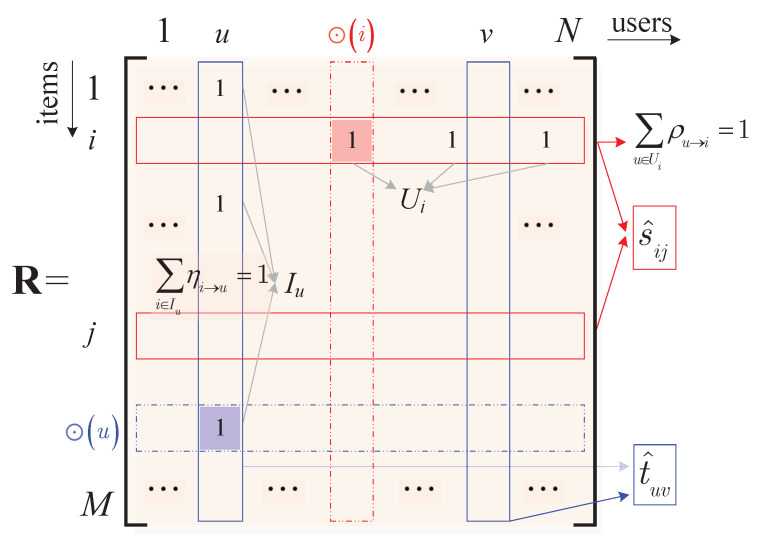
The **R** matrix and weighting coefficients η and ρ.

In order to improve the running speed of the algorithm and simplify the analytical model, we denote the user-item rating matrix of the system as an M×N matrix R with binary entries $r_{iu}\in \left\{1,0\right\}$. Specifically, the *i*-th row includes the ratings of item *i* made by all the users and the *u*-th column includes the ratings of user *u*, for all the items. Moreover, we have $r_{iu}=1$ if user *u* likes (with a relatively high rating score) item *i* and $r_{iu}=0$ if user *u* dislikes or has not observed item *i*. In the *i*-th row, we denote the set of users who like item *i* as $U_{i}$; in the *u*-th column, we denote the set of items liked by user *u* as $I_{u}$, as shown in Figure 1. In most cases, the matrix R is sparse.

Although all users in $U_{i}$ like item *i*, we note that not all them like *i* equally. Likewise, we know that the items in $I_{u}$ are not equally important to user *u* either. In this paper, therefore, we shall propose a mathematical recommendation model and the corresponding algorithm to find out the most important item for each user, as well as the most loyal and fanatical user for each item, which are referred to, respectively, as the *core item* ⊙(u) of user *u* and the *core user*⊙(i) of item *i*, as shown in Figure 1.

To this end, we shall use an M×N weighting matrix η to measure the importance of the items to the users, in which $\eta_{i\to u}={\left\{\eta \right\}}_{iu},$ $0\leq \eta_{i\to u}\leq 1$ and $\sum_{i\in I_{u}}\eta_{i\to u}=1$. By its definition, it is clear that the larger $\eta_{i\to u}$ is, the more important (to user *u*) item *i* would be. In particular, we have

(1) $$\eta_{i\to u}\left\{\begin{array}{cc} >0, & \;\mathrm{if}\;i\in I_{u}\;\left(\mathrm{equivalently}\;r_{iu}=1\right),\\ =0, & \;\mathrm{if}\;i\notin I_{u}\;\left(\mathrm{equivalently}\;r_{iu}=0\right). \end{array}\right.$$

We also present how the users like the items by an M×N weighting matrix ρ, in which $\rho_{u\to i}={\left\{\rho \right\}}_{iu}$, $0\leq \rho_{u\to i}\leq 1$, $\sum_{u\in U_{i}}\rho_{u\to i}=1$, $\rho_{u\to i}>0$ if $u\in U_{i}$ and $\rho_{u\to i}=0$ otherwise.

Note that for a given pair of users *u* and *v*, the traditional non-weighted user similarity $t_{uv}$ is defined as the number of items that they like in common. In addition, the traditional non-weighted item similarity $s_{ij}$ between items *i* and *j* is defined as the number of users who like them in common. That is,

(2) $$\begin{array}{c} t_{uv}=\sum_{i=1}^{M}r_{iu}r_{iv}\;\mathrm{and}\;s_{ij}=\sum_{u=1}^{N}r_{iu}r_{ju}. \end{array}$$

To consider the difference in users and items, we shall define the weighted similarity between users and between items as follows.

**Definition** **1.** *The weighted similarity ${\hat{t}}_{uv}$ between users u and v is defined as*

(3) $$\begin{array}{cc} {\hat{t}}_{uv} & =\sum_{i=1}^{M}r_{iu}r_{iv}\sqrt{\eta_{i\to u}}\sqrt{\eta_{i\to v}}. \end{array}$$

That is, the weighted similarity not only depends on how many common items the two users have, but also how important to them these common items are. Since we have $\eta_{i\to u}=0$ if $r_{iu}=0$ (i.e., $i\notin I_{u}$, cf. (1)), we further have

(4) $$\begin{array}{c} {\hat{t}}_{uv}=\sum_{i\in I_{u}\cap I_{v}}\sqrt{\eta_{i\to u}\eta_{i\to v}}. \end{array}$$

**Definition** **2.** *The weighted similarity between items i and j is defined as*

(5) $$\begin{array}{cc} {\hat{s}}_{ij} & =\sum_{u=1}^{N}r_{iu}r_{ju}\sqrt{\rho_{u\to i}}\sqrt{\rho_{u\to j}}. \end{array}$$

Thus, the weighted similarity between items *i* and *j* is large if many users like both of them and like them very much. Further, we have ${\hat{s}}_{ij}=\sum_{u\in U_{i}\cap U_{j}}\sqrt{\rho_{u\to i}\rho_{u\to j}}$ since we have $\rho_{u\to i}=0$ if $r_{iu}=0$ (i.e., $u\notin U_{i}$).

### 2.2. Recommendation Model and Problem Formulation

Based on the proposed weighted similarities, we shall find out the two matrices of weighting coefficients, the core users and core items of the system. After that, we shall recommend items to users similar to their core users and recommend similar items to the core item to corresponding users.

We start from an initial weighting matrix $\eta^{\left(0\right)}={\left\{\eta_{i\to u}^{\left(0\right)}\right\}}_{M\times N}.$ Based on these weighting coefficients, the weighted similarity ${\hat{t}}_{uv}^{\left(0\right)}$ between any pair of users *u* and *v* can be calculated by (4).

For an item *i*, it is noted that $U_{i}$ is the set of users who like the item *i*, and thus is a small group of similar users. When we sum the weighted similarities among them together, the total similarity would be larger if the user group $U_{i}$ is more compact (similar). Thus, we are interested in searching for such a user ${⊙}^{\left(0\right)}\left(i\right)$ that the sum of the weighted similarity between ${⊙}^{\left(0\right)}\left(i\right)$ and other users in $U_{i}$ can be maximized. In doing so, ${⊙}^{\left(0\right)}\left(i\right)$ would be the most representative user in $U_{i}$ and the most important user for item *i*, and thus is referred to as the core user of item *i*.

We denote the core user selection scheme as $S_{u}$ and denote the total weighted similarity ${\hat{\hat{T}}}_{i}^{\left(0\right)}$ centered at ${⊙}^{\left(0\right)}\left(i\right)$ as

(6) $$\begin{array}{c} {\hat{\hat{T}}}_{i}^{\left(0\right)}=\sum_{v\in U_{i}}{\hat{t}}_{{⊙}^{\left(0\right)}\left(i\right),v} \end{array}$$

which characterizes how close the users are gathered around item *i*.

Likewise, we denote the core item selection scheme as $S_{i}$ and the core item (i.e., the most representative item liked by a user) of user *u* as ${⊙}^{\left(0\right)}\left(u\right)$. The total weighted similarity of *u* centered at its core item ${⊙}^{\left(0\right)}\left(u\right)$ can be expressed as ${\hat{\hat{S}}}_{u}^{\left(0\right)}=\sum_{i\in I_{u}}{\hat{s}}_{{⊙}^{\left(0\right)}\left(u\right),i}$. Thus, ${\hat{\hat{S}}}_{u}^{\left(0\right)}$ represents how compact the item set $I_{u}$ is.

In this paper, we shall propose an iterative algorithm to solve the core users, the core items, and the weighting matrices η and ρ. In the *l*-th iteration, we denote each user-revenue by ${\hat{\hat{S}}}^{\left(l\right)}={\left[{\hat{\hat{S}}}_{1}^{\left(l\right)},{\hat{\hat{S}}}_{2}^{\left(l\right)},\cdots ,{\hat{\hat{S}}}_{N}^{\left(l\right)}\right]}^{T}$, denote each item-revenue by ${\hat{\hat{T}}}^{\left(l\right)}={\left[{\hat{\hat{T}}}_{1}^{\left(l\right)},{\hat{\hat{T}}}_{2}^{\left(l\right)},\cdots ,{\hat{\hat{T}}}_{M}^{\left(l\right)}\right]}^{T}$, and denote the *l*-th system-revenue by $d^{\left(l\right)}={\left[{\left({\hat{\hat{S}}}^{\left(l\right)}\right)}^{T},{\left({\hat{\hat{T}}}^{\left(l\right)}\right)}^{T}\right]}^{T}$. In an *L*-iteration running of the algorithm, we define the total revenue $V(\eta^{\left(0\right)})$ of the system as

(7) $$V\left(\eta^{\left(0\right)}\right)=\sum_{l=1}^{L}\alpha^{L-l}d^{\left(l\right)},$$

in which the discount factor α→0 is a small and positive number. The rationale behind the discount factor is that the revenues obtained in the past are less important than revenues obtained at present.

It is clear that as α approaches zero, the weighted revenues of the first L−1 rounds approach zero. Since we have $0^{0}=1$, the total revenue $V(\eta^{\left(0\right)})$ degrades to the revenue of the *L*-th iteration. Note that the elements ${\hat{\hat{S}}}_{u}$ and ${\hat{\hat{T}}}_{i}$ in $V(\eta^{\left(0\right)})$ are the sums of weighted similarities among users and items. Thus, ${\hat{\hat{S}}}_{u}^{\left(l\right)}$ and ${\hat{\hat{T}}}_{i}^{\left(l\right)}$ would be maximized when the correct group centers are found, i.e., the most representative items and users are found.

Next, we shall then solve the following maximization problem

(8) $$\begin{array}{c} \max_{S_{u},S_{i}}V\left(\eta^{\left(0\right)}\right) \end{array}$$

by designing the proper core user selection scheme $S_{u}$ and core item selection scheme $S_{i}$.

### 2.3. Algorithm Initialization

We propose an iterative algorithm CUIS to solve problem (8), as shown in Algorithm 1. With a certain initial weighting matrix $\eta^{\left(0\right)}$, we shall find the core users, the weighting matrix $\rho^{\left(l\right)}$, the core items, and a new weighting matrix $\eta^{\left(l\right)}$ successively and iteratively.
**Algorithm 1** The CUIS Algorithm1: **Input**: the rating matrix R, tolerable iterative error ϵ 2: **Output**: ρ: weighting matrix of users’ importance to items            η: weighting matrix of items’ importance to users            ⊙(u): vector of core item of each user            ⊙(i): vector of core user of each item 3: **Initialize**: *l* = 0, Δ=∞      Calculate ${⊙}^{\left(0\right)}\left(i\right)$ with $\eta_{i\to u}^{\left(0\right)}$ by Equation (11) 4: **While**: Δ>ϵ, **do** 5: Calculate $\rho^{\left(l\right)}$ and ${⊙}^{\left(l\right)}\left(u\right)$ by Equations (12) and (15) 6: Calculate $\eta^{\left(l\right)}$ and ${⊙}^{\left(l\right)}\left(i\right)$ by Equations (17) and (20) 7: Calculate $\Delta ={∥\eta^{\left(l+1\right)}-\eta^{\left(l\right)}∥}_{\infty }$ 8: l=l+1 9: **End While**


We begin with an initial weighting matrix $\eta^{\left(0\right)}$ in which

(9) $$\eta_{i\to u}^{\left(0\right)}=\frac{1}{\left|I_{u}\right|}$$

for each 1≤u≤N and $i\in I_{u}$. Note that $\left|I_{u}\right|$ is the number of items in $I_{u}$, i.e., the number of items user *u* likes. In a practical system, the users interact with items differently. For example, a user may watch some movies for many times and watch some movies only by some pieces. We can find a better initial weighting matrix if all these information can be exploited. In this paper, we initialize the weighting coefficients $\eta^{\left(0\right)}$ evenly for simplicity. It is also shown in Section 2.5 that the CUIS converges to the optimal solution regardless of the initial point.

According to (4), we then calculate the weighted similarity ${\hat{t}}_{uv}^{\left(0\right)}$ between each pair of users $u,v\in U_{i}$ for item *i*. For each $u\in U_{i}$, the total weighted similarity centered at *u* is

(10) $$\begin{array}{c} {\hat{T}}_{u\to i}^{\left(0\right)}=\sum_{v\in U_{i}}{\hat{t}}_{uv}^{\left(0\right)}=\sum_{v\in U_{i}}\sum_{j\in I_{u}\cap I_{v}}\sqrt{\eta_{j\to u}^{\left(0\right)}\eta_{j\to v}^{\left(0\right)}}. \end{array}$$

In particular, we use such a core user selection scheme $S_{u}$ that the core user of item *i* is chosen as the user satisfies

(11) $$\begin{array}{c} {⊙}^{\left(0\right)}\left(i\right)=\arg\max_{u\in U_{i}}\;{\hat{T}}_{u\to i}^{\left(0\right)}. \end{array}$$

As it will be proved in Section 2.5, this chosen core user selection scheme guarantees that the total revenue of the system would be maximized. Furthermore, we denote the maximum total weighted similarity achieved by item *i* as ${\hat{\hat{T}}}_{i}^{\left(0\right)}=\max_{u\in U_{i}}{\hat{T}}_{u\to i}^{\left(0\right)}={\hat{T}}_{{⊙}^{\left(0\right)}\left(i\right)\to i}^{\left(0\right)}$, i.e., the total weighted similarity centered at ${⊙}^{\left(0\right)}\left(i\right)$.

### 2.4. Update Process

Each iteration of CUIS consists of the following four steps: updating $\rho^{\left(l\right)}$, updating the core item for each user, updating $\eta^{\left(l\right)}$, and updating the core user for each item.

#### 2.4.1. Updating $\rho_{u\to i}^{\left(l\right)}$

For each item *i*, the weighting coefficient $\rho_{u\to i}^{\left(l\right)}$, i.e., the importance of a user $u\in U_{i}$ to item *i*, is evaluated by the normalized weighted similarity ${\hat{t}}_{u,{⊙}^{\left(l-1\right)}\left(i\right)}^{\left(l-1\right)}$ with the core user ${⊙}^{\left(l-1\right)}\left(i\right)$. That is,

(12) $$\begin{array}{c} \rho_{u\to i}^{\left(l\right)}=\left\{\begin{array}{cc} \frac{{\hat{t}}_{u,{⊙}^{\left(l-1\right)}\left(i\right)}^{\left(l-1\right)}}{\sum_{v\in U_{i}}{\hat{t}}_{v,{⊙}^{\left(l-1\right)}\left(i\right)}^{\left(l-1\right)}}, & u\in U_{i}\\ 0, & u\notin U_{i}. \end{array}\right. \end{array}$$

We can also present $\rho_{u\to i}^{\left(l\right)}$ in a more concise form as $\rho_{u\to i}^{\left(l\right)}={\hat{t}}_{u,{⊙}^{\left(l-1\right)}\left(i\right)}^{\left(l-1\right)}/{\hat{\hat{T}}}_{i}^{\left(l-1\right)}$. Moreover, it is seen that $\sum_{u\in U_{i}}\rho_{u\to i}^{\left(l\right)}=1$ is satisfied naturally.

#### 2.4.2. Updating Core Items

For each user *u* and each pair of items $i,j\in I_{u}$, we evaluate the weighted similarity between the two items as (cf. (5))

(13) $$\begin{array}{c} {\hat{s}}_{ij}^{\left(l\right)}=\sum_{v\in U_{i}\cap U_{j}}\sqrt{\rho_{v\to i}^{\left(l\right)}\rho_{v\to j}^{\left(l\right)}}. \end{array}$$

Centered at item *i*, the total weighted similarity ${\hat{S}}_{i\to u}^{\left(l\right)}$, i.e., the contribution (to user *u*) achieved by the item *i* is

(14) $${\hat{S}}_{i\to u}^{\left(l\right)}=\sum_{j\in I_{u}}{\hat{s}}_{ij}^{\left(l\right)}=\sum_{j\in I_{u}}\sum_{v\in U_{i}\cap U_{j}}\sqrt{\rho_{v\to i}^{\left(l\right)}\rho_{v\to j}^{\left(l\right)}}.$$

It is clear that the larger ${\hat{S}}_{i\to u}^{\left(l\right)}$ is, the more important to user *u* and the more representative the item *i* would be. Thus, we use such a core item selection scheme $S_{i}$ that the item with the largest ${\hat{S}}_{i\to u}^{\left(l\right)}$ is chosen. That is, the core item ${⊙}^{\left(l\right)}\left(u\right)$ of the user *u* is chosen as

(15) $$\begin{array}{c} {⊙}^{\left(l\right)}\left(u\right)=\arg\max_{i\in I_{u}}\;{\hat{S}}_{i\to u}^{\left(l\right)}. \end{array}$$

We denote the maximum total weighted similarity achieved by user *u* as

(16) $$\begin{array}{c} {\hat{\hat{S}}}_{u}^{\left(l\right)}=\max_{i\in I_{u}}{\hat{S}}_{i\to u}^{\left(l\right)}={\hat{S}}_{{⊙}^{\left(l\right)}\left(u\right)\to u}^{\left(l\right)}, \end{array}$$

i.e., the total weighted similarity centered at ${⊙}^{\left(l\right)}\left(u\right)$.

#### 2.4.3. Updating $\eta_{i\to u}^{\left(l\right)}$

For each user, we then quantify the importance of each of his items by the normalized weighted similarities.

(17) $$\begin{array}{c} \eta_{i\to u}^{\left(l\right)}=\left\{\begin{array}{cc} \frac{{\hat{s}}_{i,{⊙}^{\left(l\right)}\left(u\right)}^{\left(l\right)}}{\sum_{j\in I_{u}}{\hat{s}}_{j,{⊙}^{\left(l\right)}\left(u\right)}^{\left(l\right)}}, & i\in I_{u}\\ 0, & i\notin I_{u}. \end{array}\right. \end{array}$$

It is clear that $\sum_{i\in I_{u}}\eta_{i\to u}^{\left(l\right)}=1$ and the closer to ${⊙}^{\left(l\right)}\left(u\right)$ an item is, the more important to *u* it would be. Moreover, we have $\eta_{i\to u}^{\left(l\right)}={\hat{s}}_{i,{⊙}^{\left(l\right)}\left(u\right)}^{\left(l\right)}/{\hat{\hat{S}}}_{u}^{\left(l\right)}$.

#### 2.4.4. Updating Core Users

Based on the updated weighting coefficients $\eta_{i\to u}^{\left(l\right)}$, we shall re-evaluate the weighted similarity between each pair of users $u,v\in U_{i}$, for each item *i*. In doing so, we could see how similar to (or how far from) each other the users are, so that the most representative (core) user of an item could be updated. Specifically, for a certain item *i*, the weighted similarity between users $u,v\in U_{i}$ is given by (cf. (4))

(18) $$\begin{array}{c} {\hat{t}}_{uv}^{\left(l\right)}=\sum_{i\in I_{u}\cap I_{v}}\sqrt{\eta_{i\to u}^{\left(l\right)}\eta_{i\to v}^{\left(l\right)}}. \end{array}$$

Centered at user *u*, the total weighted similarity ${\hat{T}}_{u\to i}^{\left(l\right)}$, i.e., its total contribution (to item *i*) as a center is

(19) $${\hat{T}}_{u\to i}^{\left(l\right)}=\sum_{v\in U_{i}}{\hat{t}}_{uv}^{\left(l\right)}=\sum_{v\in U_{i}}\sum_{j\in I_{u}\cap I_{v}}\sqrt{\eta_{j\to u}^{\left(l\right)}\eta_{j\to v}^{\left(l\right)}}.$$

Afterwards, we use such a core user selection scheme $S_{u}$ that the user with the largest ${\hat{T}}_{u\to i}^{\left(l\right)}$ is chosen. That is,

(20) $$\begin{array}{c} {⊙}^{\left(l\right)}\left(i\right)=\arg\max_{u\in U_{i}}\;{\hat{T}}_{u\to i}^{\left(l\right)}. \end{array}$$

In particular, we denote ${\hat{\hat{T}}}_{i}^{\left(l\right)}=\max_{u\in U_{i}}{\hat{T}}_{u\to i}^{\left(l\right)}={\hat{T}}_{{⊙}^{\left(l\right)}\left(i\right)\to i}^{\left(l\right)}$.

For the proposed CUIS algorithm (cf. Algorithm 1), variables $\rho_{u\to i}^{\left(l\right)}$, ${⊙}^{\left(l\right)}\left(u\right)$, $\eta_{i\to u}^{\left(l\right)}$, and ${⊙}^{\left(l\right)}\left(i\right)$ will be updated sequentially and iteratively. In the following, we present an example of how the algorithm operates. More importantly, we shall prove that the used core user and core item selection schemes are optimal, and the CUIS algorithm is guaranteed to be convergent.

**Example** **2.** *We consider the example shown Table 1 again. By mapping the scores with 4 points and 5 points into 1 and map other ratings to 0, we have the following binary rating matrix.*

(21) $$R=\left[\begin{array}{ccccc} 1 & 1 & 1 & 0 & 0\\ 1 & 0 & 1 & 1 & 0\\ 1 & 0 & 1 & 0 & 0\\ 0 & 0 & 0 & 1 & 0\\ 0 & 0 & 0 & 1 & 1 \end{array}\right].$$

*In the simulation, we observe that CUIS has run for six iterations before it converges. For notational simplicity, we replace the names of the movies and the users with $u_{m}$ and $i_{n}$. The final weighting matrices η and ρ are shown in Table 3, with the core item ⊙(u) of each user, core user ⊙(i) of each item and the maximum total weighted similarities given in Table 4.*

*First, it is observed that item $i_{4}$ is liked only by user $u_{4}$ while user $u_{5}$ likes only one item, i.e., $i_{5}$. Thus, it is no doubt that the core user of $i_{4}$ is $u_{4}$ and the core item of $u_{5}$ is $i_{5}$, which is in accordance with Table 4. Second, although item $i_{1}$ is liked by $u_{1}$, $u_{2}$, and $u_{3}$, user $u_{2}$ is more important since $u_{2}$ likes it much more than other items, and thus is the core user of $i_{1}$. Thus, investigating the profile and the unique demands of $u_{2}$ would be useful for the system, especially for $i_{1}$. Likewise, although user $u_{3}$ like three items (i.e., $i_{1},i_{2},i_{3}$), $u_{3}$ has chosen $i_{3}$ with his special sight of items (other users pay little attention to $i_{3}$). Thus, there are good reasons to say that $i_{3}$ is important to $u_{3}$ and is his core item.*

### 2.5. Convergence Analysis

In this section, we show that the updating scheme (cf. Algorithm 1, Section 2.4) and the chosen core user/item selection schemes (cf. (11), (15), (20)) guarantee that the CUIS algorithm converges quickly.

Given an initial weighting matrix η and the corresponding total revenue V(η) (cf. (7)), we define a functional T(·) of V as follows.

(22) $$TV\left(\eta \right)=\max_{⊙(i),⊙(u)}\;\left\{\alpha V(\eta )+(1-\alpha )d(\eta )\right\},$$

in which α→0 is small positive number.

That is, in each iteration, we update V(η) to be the weighted sum of itself and the current revenue d(η), which is maximized by properly selecting the core items and core users. Note that $d={\left[{\left(\hat{\hat{S}}\right)}^{T},{\left(\hat{\hat{T}}\right)}^{T}\right]}^{T}$ and both V(η) and d(η) are N+M dimensional vectors. Thus, the maximization operation in (22) is performed element by element, i.e.,

(23) $$\begin{array}{cc} {\left(TV\left(\eta \right)\right)}_{k} & =\max_{⊙(u)}\;\left\{\alpha {\left(V\left(\eta \right)\right)}_{k}+\left(1-\alpha \right){\hat{S}}_{k}\right\}, \end{array}$$

(24) $$\begin{array}{cc} {\left(TV\left(\eta \right)\right)}_{k} & =\max_{⊙(i)}\;\left\{\alpha {\left(V\left(\eta \right)\right)}_{k}+\left(1-\alpha \right){\hat{T}}_{k-N}\right\}, \end{array}$$

for 1≤k≤N and N+1≤k≤N+M, respectively. ${\left(TV\left(\eta \right)\right)}_{k}$ and ${\left(V\left(\eta \right)\right)}_{k}$ denote the *k*-th element of the corresponding vectors. Since the previous revenue ${\left(V\left(\eta \right)\right)}_{k}$ is known and is a constant, ${\left(TV\left(\eta \right)\right)}_{k}$ would be maximized if ${\hat{S}}_{k}$ or ${\hat{T}}_{k}$ is maximized, which can be solved, respectively, by the core item selection scheme $S_{i}$ (cf. (15)) and the core user selection scheme $S_{u}$ (cf. (20)) used in CUIS. Next, we show the contraction property of the functional T(·) in the following theorem.

**Theorem** **1.** *The functional TV(η) defined in (22) is a contraction mapping under the infinite norm.*

**Proof.**  We denote u(η) and v(η) as two revenue functions of the weighting matrix η and denote them as u and v for notational simplicity. We have,

(25) $$\begin{array}{cc} T(u) & =\max_{⊙}\;\left\{\alpha u+(1-\alpha )d\right\}\\ \; & =\max_{⊙}\;\left\{\alpha v+\alpha (u-v)+(1-\alpha )d\right\}\\ \; & \leq \max_{⊙}\;\left\{\alpha v+(1-\alpha )d\right\}+\alpha {∥u-v∥}_{\infty }\cdot 1\\ \; & =T\left(v\right)+\alpha {∥u-v∥}_{\infty }\cdot 1, \end{array}$$

in which 1 is an all-ones vector, the inequality holds because each element of α(u−v) is no larger than the largest element of itself. Thus, we have

(26) $$T\left(u\right)-T\left(v\right)\leq \alpha {∥u-v∥}_{\infty }\cdot 1.$$

Likewise, we also have

(27) $$T\left(v\right)-T\left(u\right)\leq \alpha {∥u-v∥}_{\infty }\cdot 1.$$

By combining (26) and (27), we finally have

(28) $${∥T(u)-T(v)∥}_{\infty }\leq \alpha {∥u-v∥}_{\infty },$$

which means that functional TV(η) is a contraction mapping under the infinite norm. This completes the proof. □

According to Banach’s fixed point theorem [34], by applying the contraction mapping operator T(·) repeatedly to an initial point $\eta^{\left(0\right)}$, the proposed CUIS algorithm is guaranteed to converge, and the output core users, core items would maximize the total revenue $V(\eta^{\left(0\right)})$.

## 3. CUIS Based Recommenders

### 3.1. Recommendation Methods

With the CUIS algorithm, we have obtained the core user ⊙(i) of each item *i*, the core item ⊙(u) of each user *u*, the optimal weighting matrix η indicating the importance of items to users, and the optimal weighting matrix ρ indicating the importance of users to items. In this section, we shall make recommendations for users based on these obtained results. In particular, we can either find and recommend a proper item to a user or find a proper user for an item, which are referred to, respectively, as the *core item* (CI) based recommender and the *core user* (CU) based recommender.

#### 3.1.1. Core User and Core Item Based Recommenders

In a *CU-recommender*, we try to use the obtained information of core users. To be specific, we calculate the weighted user similarity ${\hat{t}}_{⊙\left(i\right),v}$ between the core user ⊙(i) and each user *v* except the core user following (3). Then, we shall recommend item *i* to the user with the largest weighted similarity ${\hat{t}}_{⊙\left(i\right),v}$.

Likewise, we try to use the obtained information of core items in a *CI-recommender*. That is, we calculate the weighted item similarity ${\hat{s}}_{⊙\left(u\right),j}$ between the core item ⊙(u) and each item *j* except the core item following (5). Then, we shall recommend the item with the largest weighted similarity ${\hat{s}}_{⊙\left(u\right),j}$ to user *u*.

#### 3.1.2. Core User and Core Item Based *k*-Means Clustering Recommenders

We note that finding the proper item out of all the items or finding a proper user out of all the users is very computing consuming. Thus, we shall combine the CU-recommender and the CI-recommender with the *k*-means clustering method, which are referred to, respectively, as the *CU-kMCL-recommender* and the *CI-kMCL-recommender*.

In a *CU-kMCL-recommender*, we first group all the users into *k* clusters using the *k*-means method. In particular, the traditional similarity measure is replaced by the weighted user similarity measure of this paper, as shown in (3). For a given item *i*, we then find the most similar user $u^{*}$ with its core user ⊙(i) (i.e., with the largest ${\hat{t}}_{u⊙(i)}$) within the cluster in which ⊙(i) belongs to. Next, item *i* is recommended to user $u^{*}$.

In a *CI-kMCL-recommender*, we group all the items into *k* clusters using the *k*-means method and calculate item similarity with the weighted item similarity measure (cf. (5)). For a given user *u*, we then find the most similar item $i^{*}$ with its core item ⊙(u) (i.e., with the largest ${\hat{s}}_{i⊙(u)}$) within the cluster in which ⊙(u) belongs to. Next, item $i^{*}$ is recommended to user *u*.

In doing so, the CU-*k*MCL-recommender and CI-*k*MCL-recommender not only find out your best “friend” from your neighborhood and recommend his “lobster” to you, but also save memory space, reduce computing time and release scalability issues.

#### 3.1.3. Weighted Similarity Based PAF Recommenders

The proposed weighted similarity can also be combined with the popularity amongst friends (PAF) recommender [35]. It can also be implemented in two modes, which are referred to, respectively, as the weighted user similarity based PAF recommender (*WUS-PAF-recommender*) and the weighted item similarity based PAF recommender (*WIS-PAF-recommender*).

In the traditional PAF method, the top-*F* best fiends (denoted as set $F_{\mathrm{Eu},u}^{\mathrm{user}}$) of each user *u* would be found out based on non-weighted similarity measure (cf. (2)). For each item, we then calculate the number $p_{R,i}$ of recommendations (i.e., $r_{iv}=1$) made by these best friends. That is,

(29) $$\begin{array}{c} p_{R,i}=\sum_{v\in F_{\mathrm{Eu},u}^{\mathrm{user}}}r_{iv},\;\forall \;i\in I, \end{array}$$

in which I={1,2,⋯,M} is the set of all items. The item $i_{R}^{*}$ with the largest $p_{R,i}$ is considered as the most popular amongst the friends of user *u*, and thus is recommended to user *u*. Namely, the recommended item satisfies

(30) $$\begin{array}{c} i_{R}^{*}=\arg\max_{i\in I}p_{R,i}. \end{array}$$

The weighted similarity based PAF recommenders try to improve performance by considering the difference among users and items.

**Figure 2:**
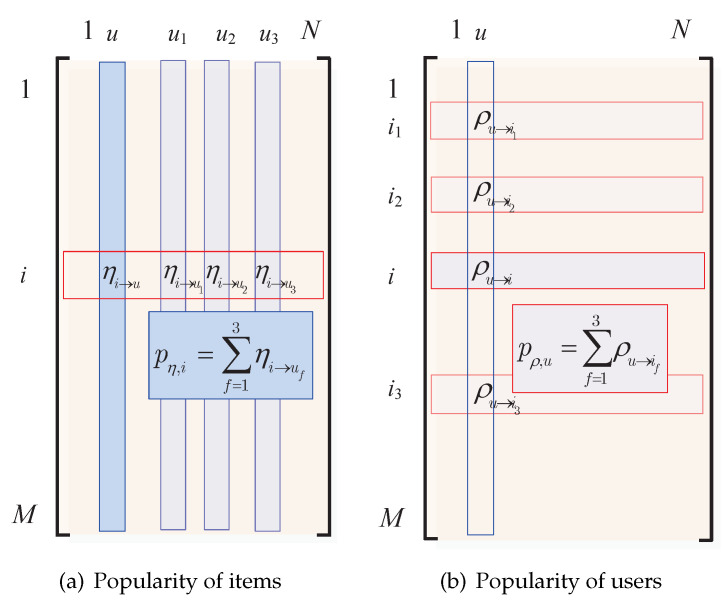
Popularity calculations with weighting matrices η and ρ, respectively, Formulas (31) and (33) have been simplified under binary rating matrix. Suppose user *u* has 3 friends $u_{1},u_{2},u_{3}$ and item *i* has 3 friends $i_{1},i_{2},i_{3}$.

To be specific, a *WUS-PAF-recommender* identifies the top-*F* best fiends (denoted as set $F_{\mathrm{ws},u}^{\mathrm{user}}$) of a user *u* through their weighted similarities (cf. (3)) instead of the non-weighted user similarity. As mentioned in Section 2.2 and Equation (1), the items liked by a user *u* (i.e., $i\in I_{u}$) are not equally important to *u*. Thus, we say that an item *i* is popular among friends if many friends like *i* and *i* is very important to them (i.e., with large $\eta_{i\to u}$). To be specific, the popularity of an item based on weighting matrix η is defined as (cf. Figure 2a)

(31) $$\begin{array}{c} p_{\eta ,i}=\sum_{v\in F_{\mathrm{ws},u}^{\mathrm{user}}}r_{iv}\eta_{i\to v},\;\forall \;i\in I, \end{array}$$

and the item $i_{\eta }^{*}$ with the largest $p_{\eta ,i}$ is recommended to user *u*. Namely, the recommended item satisfies

(32) $$\begin{array}{c} i_{\eta }^{*}=\arg\max_{i\in I}p_{\eta ,i}. \end{array}$$

Likewise, a *WIS-PAF-recommender* would find out the friends of an item and recommend this item to the user that in favor of these similar items most. First, we identify the top-*F* best item friends (denoted as set $F_{\mathrm{ws},i}^{\mathrm{item}}$) of an item *i* through their weighted similarities (cf. (5)). We say that a user *u* is popular to $F_{\mathrm{ws},i}^{\mathrm{item}}$ if *u* like many of these items and *u* is very important to these items (i.e., with large $\rho_{u\to i}$). To be specific, the popularity of a user based on weighting matrix ρ is defined as (cf. Figure 2b)

(33) $$\begin{array}{c} p_{\rho ,u}=\sum_{j\in F_{\mathrm{ws},i}^{\mathrm{item}}}r_{ju}\rho_{u\to j},\;\forall \;u\in U, \end{array}$$

and the user $u_{\rho }^{*}$ with the largest $p_{\rho ,u}$ is recommended to item *i*:

(34) $$\begin{array}{c} u_{\rho }^{*}=\arg\max_{u\in U}p_{\rho ,u}, \end{array}$$

in which U={1,2,⋯,N} is the set of all users.

We can also understand the PAF recommenders from the perspective of the core users and the core items. Take the *WUS-PAF-recommender* as an example, we consider all the neighbors of the target user as an virtual user, then the most popular item amongst these neighbors can be viewed as the core item of this virtual user. Therefore, the recommendation process of *WUS-PAF-recommender* can be understood as follows. First, we find the virtual user closest to the target user (i.e., user neighbors). Second, we recommend the “core item” of the virtual user (the most popular item amongst the user neighbors) to the target user. Likewise, in the *WIS-PAF-recommender*, we treat all neighbors of the target item as an virtual individual item. We treat the most loyal user amongst the item neighbors as the core user of this virtual item. Thus, the recommendation process can be regarded as finding the virtual item closest to the target item (i.e., item neighbors) first, and then recommending the target item to the “core user” of this virtual item (the most loyal user amongst item neighbors).

### 3.2. Computational Complexity

The recommendation process of this paper includes two stages, namely the knowledge discovery stage (CUIS, cf. Algorithm 1) and the recommendation generation stage. In this section, we denote the number of items as *m*, the number of users as *n*, the non-zeros in recommendation matrix R as nnz, the number of CUIS iterations as *l*, the number of clusters as *k*, and the number of *k*-means iterations as $l_{k}$. Note that matrices ρ, η and R have the same number of non-zeros.

In the initialization phase of CUIS, the calculation complexity of $\eta^{\left(0\right)}$ is O(nnz∗2). Assuming that for each item *i*, the average length of $U_{i}$ is $d_{\left|U_{i}\right|}$, i.e., $d_{\left|U_{i}\right|}=\mathrm{nnz}/m$, and for each user *u*, the average length of $I_{u}$ is $d_{\left|I_{u}\right|}$, i.e., $d_{\left|I_{u}\right|}=\mathrm{nnz}/n$. The complexity of calculating a ${\hat{T}}_{u\to i}^{\left(0\right)}$ is $O(d_{\left|U_{i}\right|}\cdot m)$, thus the complexity of calculating all ${\hat{T}}_{u\to i}^{\left(0\right)}$ is $O(\mathrm{nnz}\cdot d_{\left|U_{i}\right|}\cdot m)$. After that, the complexity of obtaining ${⊙}^{\left(0\right)}\left(i\right)$ of all items is O(nnz). Therefore, the computational complexity of the initialization phase is $O(\mathrm{nnz}d_{\left|U_{i}\right|}m+3\mathrm{nnz})$, and can also be simplified as $O({\mathrm{nnz}}^{2})$.

The complexity of updating all numerators of $\rho^{\left(l\right)}$ (cf. (12)) is O(nnz·m), while the complexity of the operation of sum in denominators and dividing the numerators by the denominators are both O(nnz). The complexity of calculating a ${\hat{S}}_{i\to u}^{\left(l\right)}$ is $O(d_{\left|I_{u}\right|}\cdot n)$, thus the complexity of calculating all ${\hat{S}}_{i\to u}^{\left(l\right)}$ is $O(\mathrm{nnz}\cdot d_{\left|I_{u}\right|}\cdot n)$. After that, the complexity of obtaining ${⊙}^{\left(l\right)}\left(u\right)$ of all users is O(nnz). Therefore, the computational complexity for updating $\rho^{\left(l\right)}$ and ${⊙}^{\left(l\right)}\left(u\right)$ is $O(\mathrm{nnz}\left(m+d_{\left|I_{u}\right|}n\right)+3\mathrm{nnz})$, and can be simplified as O(nnz(m+nnz)).

The complexity of updating all numerators of $\eta^{\left(l\right)}$ (cf. (17)) is O(nnz·n), while the complexity of the operation of sum in denominators and dividing the numerators by the denominators are both O(nnz). The complexity of calculating all ${\hat{T}}_{u\to i}^{\left(l\right)}$ is $O(\mathrm{nnz}\cdot d_{\left|U_{i}\right|}\cdot m)$, and then the complexity of obtaining ${⊙}^{\left(l\right)}\left(i\right)$ of all items is O(nnz). Therefore, the computational complexity for updating $\eta^{\left(l\right)}$ and ${⊙}^{\left(l\right)}\left(i\right)$ is $O(\mathrm{nnz}\left(n+d_{\left|U_{i}\right|}m\right)+3\mathrm{nnz})$, and can be simplified as O(nnz(n+nnz)).

In a finite number *l*-iteration running, the overall computational complexity of CUIS can be approximately expressed as $O({\mathrm{nnz}}^{2}+\mathrm{nnz}\left(m+n+2\mathrm{nnz}\right)l)$. Since the algorithm is guaranteed to converge in a few number of iterations, *l* can be considered as a finite constant. Thus, the overall computational complexity of CUIS degrades to $O({\mathrm{nnz}}^{2})$.

In the recommendation generation stage, we characterize the relationships among users by an undirected graph, in which an edge exists between vertex *u* and *v* iff ${\hat{t}}_{uv}>0$. We denote the average degree of vertexes in the graph as $\bar{d_{u}}$. Likewise, we express the relations among items with another graph, in which an edge exists between vertex *i* and *j* iff ${\hat{s}}_{ij}>0$, we denote the average degree item vertexes in the graph as $\bar{d_{i}}$. For recommenders with *k*-means clustering, the users or items are grouped in to *k* clusters while the graph is divided into *k*-sub-graphs, and the corresponding complexity are expressed as $O(nkl_{k})$ and $O(mkl_{k})$, respectively. We denote corresponding average degree of each user and each item, respectively, as $\bar{d_{\hat{u}}}$ and $\bar{d_{\hat{i}}}$. It is noted that $\bar{d_{u}}$, $\bar{d_{i}}$, $\bar{d_{\hat{u}}}$ and $\bar{d_{\hat{i}}}$ are generally smaller than *m* or *n*. Thus, the overall complexity of the recommendation stage is low. Detailed information on the complexity of CUIS and the proposed recommenders are summarized in Table 5.

## 4. Experiments

In this section, we investigate the performance of the proposed CUIS algorithm (cf. Algorithm 1) and recommenders on two real datasets. Particularly, we try to answer the following two questions: (1) does CUIS algorithm converge on the real data sets and how fast does it converge? (2) whether the proposed recommenders can improve the accuracy of recommendations compared with the traditional non-weighted similarity based recommenders? The purpose of our experiments is not to compare our methods with excellent state-of-the-art recommendation solutions, but to illustrate the weighting coefficients can be combined with many similarity measures and show that the weighted similarity measure improves the performance of recommendations.

### 4.1. Experiment Setup

#### 4.1.1. Datasets

In the experiments, we use the following two real-world movie datasets, i.e., *MovieLens100k* and *MovieLens1m*, which consist of, respectively, $10^{5}$ ratings and $10^{6}$ ratings [36], and denote them as ML100k and ML1m. The overall statistics of the two datasets are presented in Table 6 and the datasets are available online (https://grouplens.org/datasets/movielens/, accesseed on 22 April 2022).

In the original datasets, each user rates at least 20 of the movies with integer scores between 1 and 5. To apply the CUIS algorithm, the datasets are pre-processed as follows. First, all the ratings with 4 points and 5 points are considered as positive feedbacks and are denoted as “1” (i.e., these users are interested in the movies). The other ratings are considered as negative feedbacks and are denoted as “0” (i.e., these users dislike or have unobserved the movies). Second, the all-zeros rows and all-zeros columns are removed. In this way, we have obtained a rating matrix **R** with binary elements.

Since CUIS converges quickly, we set the maximum number of iterations to 20. In the recommendation generation stage, we shall recommend *K* items to each user or recommend each item to *K* users. Since the number *K* of recommendations affects the performance a lot, we shall implement the CUIS-based recommenders with several *K* values. For the *k*-Means clustering recommenders, we set the size *k* of the clusters to {1, 5, 10, 15, 20, 25} for the ML100k dataset and {1, 10, 20, 30, 40, 50} for the ML1m dataset. In doing so, we could see whether the size of the clusters affects the recommendation performance.

#### 4.1.2. Evaluation Metrics

For binary ratings based recommenders, we recommend *K* items to each user *u*, and evaluate the performance by recommendation error rates, which are defined, respectively, as

(35) $${\hat{\varepsilon }}_{u}=\frac{\;\#\mathrm{error}}{N\times K},$$

(36) $${\hat{\varepsilon }}_{i}=\frac{\;\#\mathrm{error}}{M\times K},$$

in which #error is the number of known wrong recommendations. We cannot judge whether the recommendations are right or wrong if the corresponding ratings of the users (or items) have never been observed. Therefore, we use the estimated value $\hat{\varepsilon }$ which only considers the number of those known recommendation errors.

Likewise, we also evaluate the performance of recommenders by right rate $\hat{\gamma }$ which are defined, respectively, as

(37) $${\hat{\gamma }}_{u}=\frac{\;\#\mathrm{right}}{N\times K},$$

(38) $${\hat{\gamma }}_{i}=\frac{\;\#\mathrm{right}}{M\times K},$$

in which #right is the number of known right recommendations.

Note that the ratings that the recommended users or items have not been observed by the system, we can’t tell whether these recommendations are right or wrong. Thus, the true recommendation error rate ε and right rate γ are higher than the estimated values $\hat{\varepsilon }$ and $\hat{\gamma }$.

In addition, the Precision and Recall are two widely used metrics to evaluate the accuracy of recommendation algorithms. To evaluate the precision and recall of the proposed CUIS based recommenders, we present the corresponding confusion matrix, as shown in Table 7.

Specifically, the precision returns the proportion of relevant items relative to all recommended items and the recall shows the proportion of relevant items relative to all actual relevant items. The expression of Precision and Recall are shown as follows:

(39) $$\mathrm{Precision}=\frac{\;\#\mathrm{TP}}{\;\#\mathrm{TP}+\;\#\mathrm{FP}},$$

(40) $$\mathrm{Recall}=\frac{\;\#\mathrm{TP}}{\;\#\mathrm{TP}+\;\#\mathrm{FN}}.$$

Moreover, we evaluated the ranking of relevant items in the recommendation list using normalized discounted cumulative gain (NDCG) and mean reciprocal rank (MRR), which respectively defined as:

(41) $$\mathrm{NDCG}=\frac{1}{\left|U\right|}\sum_{u\in U}\frac{\log2}{\log({\mathrm{rank}}_{u}+1)},$$

(42) $$\mathrm{MRR}=\frac{1}{\left|U\right|}\sum_{u\in U}\frac{1}{{\mathrm{rank}}_{u}},$$

in which U is the set of all users and ${\mathrm{rank}}_{u}$ is the hit position for the recommendation list of user *u*.

The cut-off point *K* is used for the above mentioned four metrics, in which Precision@*K* and Recall@*K* focus on how many relevant items are included in the first *K* recommended items, while NDCG@*K* and MRR@*K* account for the ranked position of relevant items of the first *K* recommended items. For all of the four evaluation metrics, the higher value indicates the better performance. In particular, we calculate the metrics with K=5 and K=20.

#### 4.1.3. Recommenders under Test

Based on the obtained information on ⊙(i), ⊙(u), η, and ρ obtained by CUIS, we consider the performance of the following recommenders:

- the *CU-recommender* and the *CI-recommender* (cf. Section 3.1.1);
- the *CU-kMCL-recommender* and the *CI-kMCL-recommender* (cf. Section 3.1.2);
- the *WUS-PAF-recommender* and the *WIS-PAF-recommender* (cf. Section 3.1.3).

In particular, each recommender can be performed either by using traditional non-weighted similarity measures (cf. (2)) or through the proposed weighted similarity measures given in (3) and (5). When the traditional similarity measure is used, the recommender will be labeled with an extra subscript ${}_{R}$. When the weighted similarity measure is used, the recommender will be labeled with an extra subscript ${}_{\eta }$ or ${}_{\rho }$.

### 4.2. Convergence

**Figure 3:**
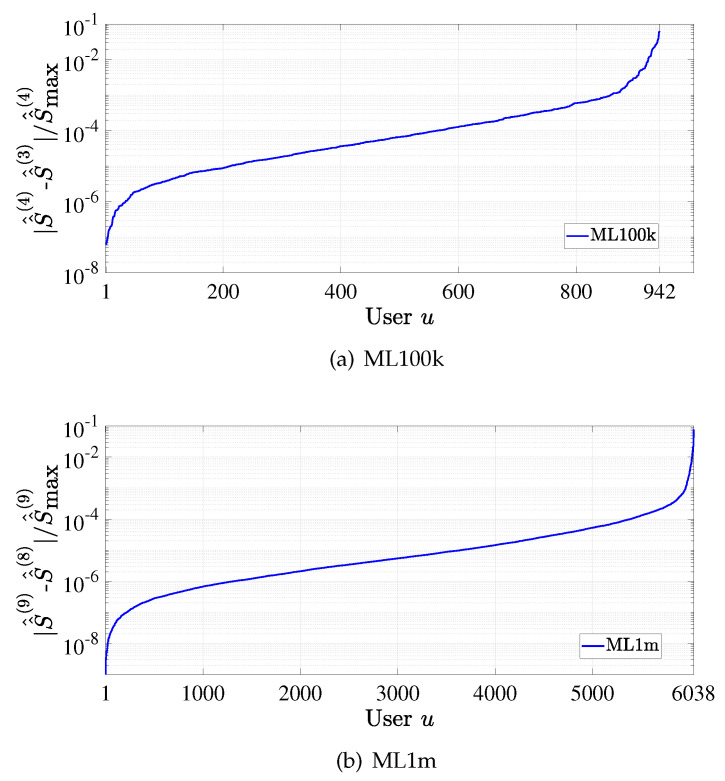
Convergence of CUIS.

To show the convergence of CUIS, we evaluate the change in the total weighted similarity ${\hat{\hat{S}}}_{u}^{\left(l\right)}$ (cf. (16)) in adjacent iterations for each user. We calculate the ratio between $|{\hat{\hat{S}}}_{u}^{\left(l\right)}-{\hat{\hat{S}}}_{u}^{\left(l-1\right)}|$ and $\max_{u}\left\{{\hat{\hat{S}}}_{u}^{\left(l\right)}\right\}$ for each user and sort them in ascending order. From the result on the ML100k dataset as shown in Figure 3a, we see that ${\hat{\hat{S}}}_{u}^{\left(l\right)}$ becomes stable for most users in only four iterations. Similar conclusion can also be drawn from the result for ML1m dataset, i.e., Figure 3b. Thus, it is clear that CUIS yields fast convergence.

### 4.3. Recommendation Error Rate

To show the effectiveness of the proposed weighted similarity, we shall compare the error performances of each recommender when the traditional similarity (cf. (2)) and the weighted similarity ((3) or (5)) are used.

**Figure 4:**
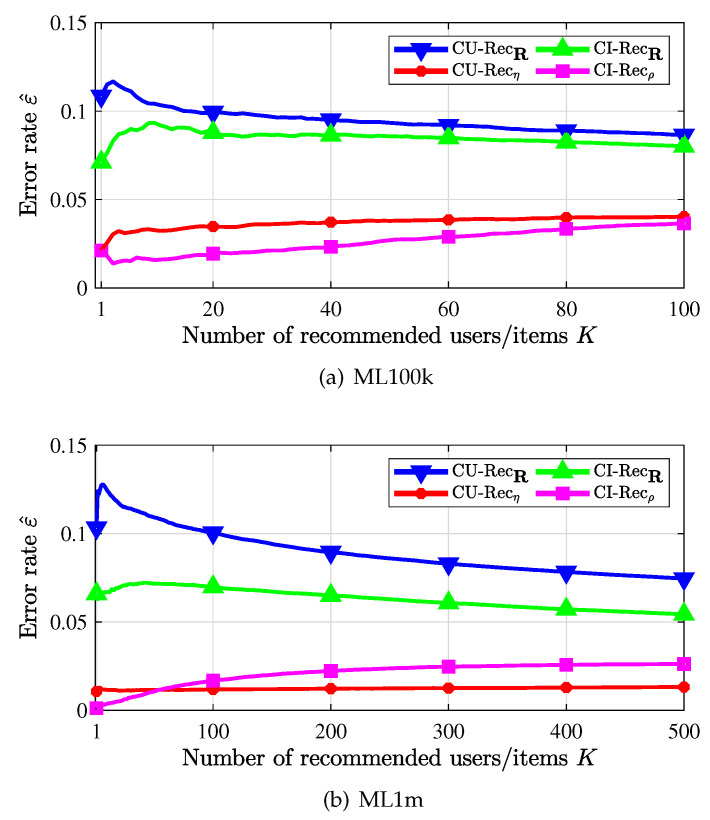
Recommendation error rate vs. number of recommended items/users.

Figure 4 presents the error rate of the CU-recommender and the CI-recommender, in which we recommend each item to top-*K* similar users with its core user or recommend top-*K* similar items with the core item to each user. First, it is observed that the error is much smaller when weighted similarity (curves labeled by * and □) is used than when the traditional similarity is used (curves labeled by ▽ and Δ). Thus, the proposed weighted similarity is reasonable and useful. Second, it is seen that the core item based CI-recommender performs better than the core user based CU-recommender. This is due to the structure of the data sets, in which each user has at least 20 ratings, while some items may have very small number of or even no users to like them. That is, the ratings for an item are much sparser than the ratings of a user, and thus very few scores can be exploited to find the core user of an item. Therefore, it would be more accurate to find the core item of a user and then its similar items for this user; the recommendation matrix is not so sufficient for looking for the core user of an item, as well as for looking for potential users to recommend. Third, for the CU-recommender, when recommending more users for items, since the ratings for items are more sparse, the estimated error rate shown in Figure 4b does not vary widely.

**Figure 5:**
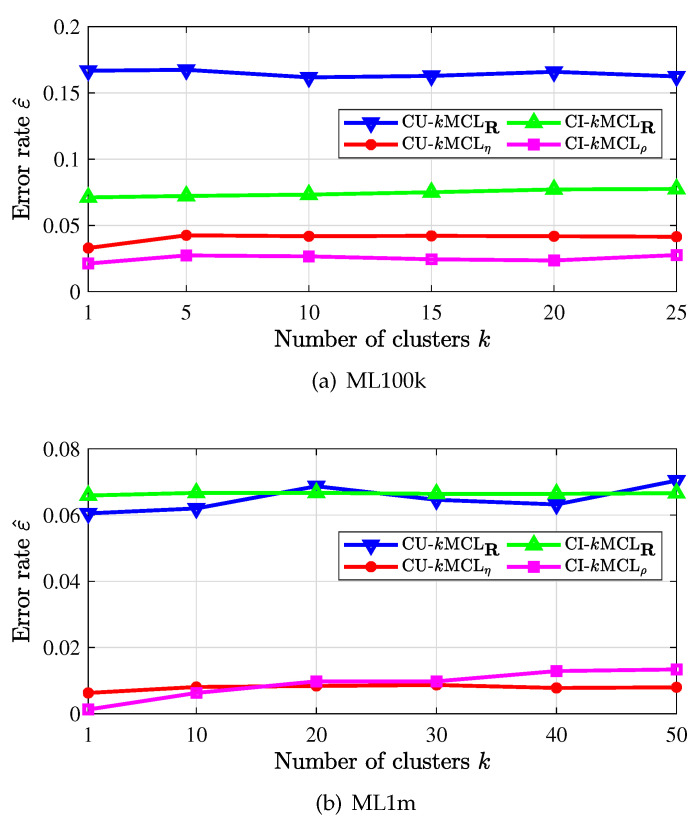
Recommendation error rate with *k*-means clustering.

Figure 5 depicts the error rate of recommenders with *k*-means clustering. By clustering users or items into *k*-clusters, searching the most similar user/item within each cluster, and then making recommendations, the computational complexity of the recommender would be largely reduced. In particular, we only need to run the clustering process once and in an offline manner. It is observed in the figures that the recommendation error rate is also smaller when the weighted similarity measures (with subscript ${}_{\eta }$ or ${}_{\rho }$) are applied, and the error rate does not change much as *k* is increased. Note that we did not run the recommenders with too much clustering due to the complexity of the clustering process. As the number *k* of clusters grows large, it is expected that the error rate would be larger, since the searching space for the right item/user is small.

**Figure 6:**
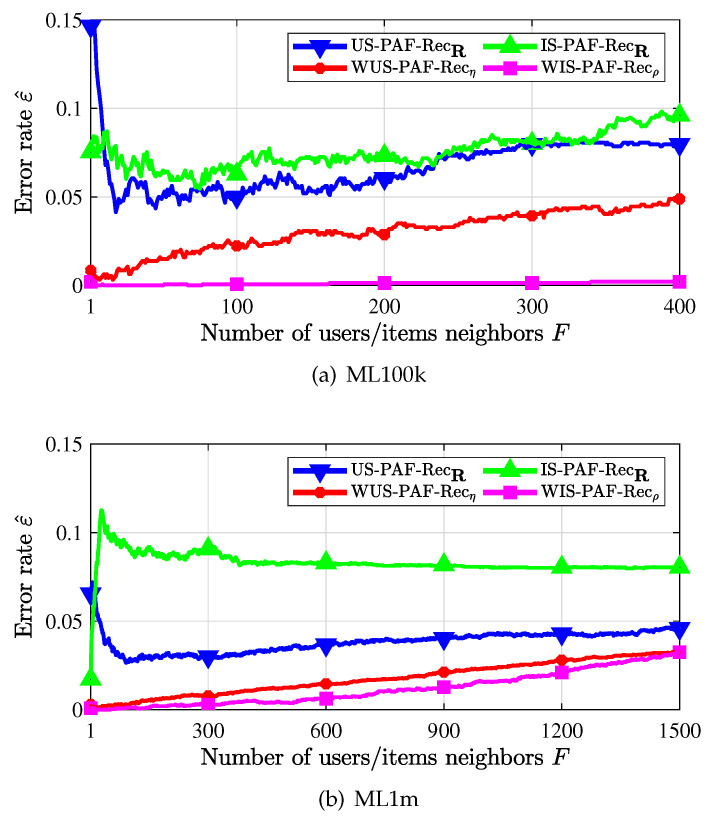
Recommendation error rate when PAF scheme is used.

The performance of CUIS plus PAF recommenders, i.e., the WUS-PAF-recommender and the WIS-PAF-recommender are presented in Figure 6. We denote the non-weighted user similarity and the non-weighted item similarity based PAF recommenders as US-PAF-Rec and IS-PAF-Rec respectively. First, we observe that recommenders using the weighted similarity measure (with subscript ${}_{\eta }$ or ${}_{\rho }$) outperform recommenders using the non-weighted similarity measure (with subscript ${}_{R}$). Second, we observe that WIS-PAF-recommender outperforms WUS-PAF-recommender, since each user has at least 20 favorable items while most items are probably liked by only a few users. Thus, it would be more accurate to find the most popular users than to find the most popular items. Third, it is seen that the error rate is large when *F* is too small or too large for US-PAF-recommender at the non-weighted similarity. In fact, too few neighbors do not ensure including most of good neighbors while too many neighbors bring in bad neighbors. This phenomenon is caused by the inaccuracy of the similarity, and we see that under the weighted similarity, the recommended error rate gradually increases with the introduction of neighbors with worse and worse similarity.

### 4.4. Recommendation Right Rate

**Figure 7:**
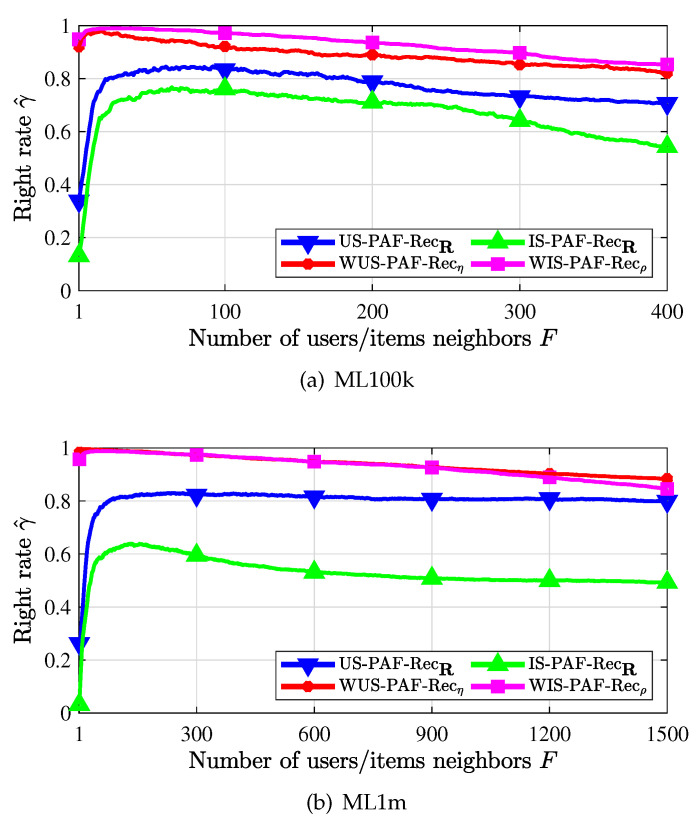
Recommendation right rate when PAF scheme is used.

We have studied the three kinds of recommenders in terms of the recommendation error rate. In this section, we evaluate the performance of the PAF recommenders from the perspective of the recommendation right rate. The comparison of the recommendation right rates between the traditional PAF recommenders and the weighted PAF recommenders is shown in Figure 7. In Figure 7a, we first observe that recommenders using the weighted similarity measure (with subscript ${}_{\eta }$ or ${}_{\rho }$) outperform the recommenders using the non-weighted similarity measure (with subscript ${}_{R}$). Second, the recommendation right rate of the weighted PAF recommenders decreases gradually when more and more neighbors that are not very similar are used and found. Third, we observe that the recommendation right rate $\hat{\gamma }$ is very high in a small number of neighbors when the weighted PAF recommenders are used. In addition, the true recommendation right rate γ is higher than the estimated value $\hat{\gamma }$. Therefore, it is observed that the weighted PAF recommenders improve the recommendation performance significantly. This also verifies the rationality and validity of the proposed weighting coefficients and weighted similarity measures. Similar results are also obtained in Figure 7b. We do not present the performance of the other two types of weighted recommenders (cf. Section 3.1.1 and Section 3.1.2) since the corresponding improvement over corresponding traditional recommenders is not very significant. One possible explanation might be that the core user or core item based recommendations inevitably limit the diversity of recommendations while the sparsity of the rating matrix R makes it difficult to determine the correctness of all the recommendations.

### 4.5. Performance Comparison with Existing Weighting Schemes

We compare the proposed weighting schemes to two commonly used weighting schemes, i.e., the variance weighting (VW) [7] and the inverse user frequency (IUF) [6], in terms of the following four metrics: Precision, Recall, NDCG and MRR.

The obtained results are listed in Table 8 and Table 9, together with the results for traditional method without using any weighting scheme, and the metrics are compared using the proposed recommenders for recommending 5 and 20 items (or users) on the ML100k dataset, respectively. For the PAF-based recommenders, we fix the number of neighbors to 20. The legends “NO”, “VW”, “IUF” and “CUIS” refer to the no weighting scheme, the variance weighting scheme, the inverse user frequency for weighting scheme and our proposed new weighting scheme CUIS. First, it is observed that the performance of the proposed CUIS weighting scheme is superior to the other three weighting schemes for all evaluation metrics, which verifies that the proposed weighting scheme can improve the accuracy of similarity and improve the quality of recommendation. Second, the variance weighting and the inverse user frequency weighting perform even worse than the non-weighted scheme under some metrics. This is actually consistent with the findings in [5,7]. Third, we observe that the metrics of CU-Rec and CI-Rec are significantly lower than those of WUS-PAF-Rec and WIS-PAF-Rec. This is because the CU-Rec and CI-Rec generate recommendations based on core users and core items. That is, only similar neighbors to core users and core items are recommended, which inevitably results in a single recommendation, and thus the system may not have many users or items similar to specific core users and core items, resulting in lower actual recommendation accuracy. Since CU-*k*MCL-Rec and CI-*k*MCL-Rec need to cluster users and items to narrow down the search range. Although some computational complexity is reduced, it leads to a decrease in recommendation accuracy. Considering that the recommended accuracy of CU-Rec and CI-Rec is moderate and the recommended accuracy of CU-*k*MCL-Rec and CI-*k*MCL-Rec is worse, they are not presented in the table.

### 4.6. CUIS with Continuous Ratings

#### 4.6.1. Weighted Similarity Measure

In this section, we extend the the weighted similarity measure and the CUIS algorithm to recommendation systems with continuous ratings. In this case, the available sets $U_{i}$ and $I_{u}$ are defined, respectively, as the set of users who has rated item *i* and the set of items rated by user *u*. Although the computational time will increase due to more refined scores, the weighted similarities among users and items would also be more precise. To show the generality and efficiency of CUIS, we combine the weighting coefficients with the adjusted cosine similarity [12]. The adjusted cosine similarity between items *i* and *j* is given by

(43) $$\begin{array}{c} s_{ij}=\frac{\sum_{u\in U_{ij}}\left(r_{iu}-\bar{r_{u}}\right)\left(r_{ju}-\bar{r_{u}}\right)}{\sqrt{\sum_{u\in U_{ij}}({r_{iu}-\bar{r_{u}})}^{2}}\sqrt{\sum_{u\in U_{ij}}{\left(r_{ju}-\bar{r_{u}}\right)}^{2}}}, \end{array}$$

in which $U_{ij}$ is the set of users who have rated both *i* and *j*, $\bar{r_{u}}$ is the average rating score of user *u*. Due to the different preferences of users (to items) and the different biases of items (to users), we shall combine the weighting coefficients ${\left\{\rho \right\}}_{iu}$ obtained by CUIS with (43), and express ${\hat{s}}_{ij}$ as

(44) $$\begin{array}{c} {\hat{s}}_{ij}=\frac{\sum_{u\in U_{ij}}\left(\rho_{u\to i}r_{iu}-\bar{\rho_{u}r_{u}}\right)\left(\rho_{u\to j}r_{ju}-\bar{\rho_{u}r_{u}}\right)}{\sqrt{\sum_{u\in U_{ij}}{\left(\rho_{u\to i}r_{iu}-\bar{\rho_{u}r_{u}}\right)}^{2}}\sqrt{\sum_{u\in U_{ij}}{\left(\rho_{u\to j}r_{ju}-\bar{\rho_{u}r_{u}}\right)}^{2}}}, \end{array}$$

in which $\rho_{u\to i}$ is the importance of user *u* to item *i*, $\bar{\rho_{u}r_{u}}$ represents the average weighted ratings of user *u*.

**Figure 8:**
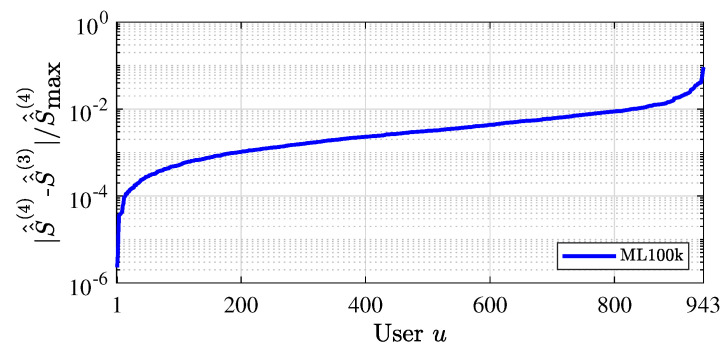
Convergence of CUIS with continuous scores.

For the CUIS with continuous scores, we shall calculate $|{\hat{\hat{S}}}_{u}^{\left(l\right)}-{\hat{\hat{S}}}_{u}^{\left(l-1\right)}|$ for each user and sort them in ascending order. From the result on the ML100k dataset as shown in Figure 8, we observe that the ${\hat{\hat{S}}}_{u}^{\left(l\right)}$ of all of the users become stable in only 4 iterations. That is, CUIS also converges quickly for systems with continuous ratings.

#### 4.6.2. Rating Prediction

In some scenarios, the users also want to know how much they will like the items we are planning to recommend. Thus, it is necessary to make rating prediction for the users rather than directly recommend items to them and tell them that “you will be interested in these items”. In item-based CF (Item CF) method [12], the prediction on an item *i* for a user *u* is defined as the weighted sum of the rating scores of the items similar to *i*, in which the weighting coefficients are chosen as the corresponding similarity $s_{ij}$ between items *i* and *j*. In this paper, we shall replace the weighting coefficients by the weighted similarity ${\hat{s}}_{ij}$ (cf. (44)) proposed in this paper. To be specific, the predicted rating ${\hat{r}}_{iu}$ is defined as

(45) $$\begin{array}{c} {\hat{r}}_{iu}=\frac{\sum_{j\in N_{i}}{\hat{s}}_{ij}r_{ju}}{\sum_{j\in N_{i}}{\hat{s}}_{ij}}, \end{array}$$

in which $N_{i}$ is the set of neighboring items similar to *i*.

For the rating prediction with continuous ratings, we verify the effectiveness of the proposed weighted similarity measure with the following two widely used evaluation metrics, i.e., the mean absolute error (MAE) and the root mean square error (RMSE),

(46) $$MAE=\frac{1}{\left|D_{\mathrm{test}}\right|}\sum_{\left(i,u\right)\in D_{\mathrm{test}}}\left|r_{iu}-{\hat{r}}_{iu}\right|,$$

(47) $$RMSE=\sqrt{\frac{1}{\left|D_{\mathrm{test}}\right|}\sum_{\left(i,u\right)\in D_{\mathrm{test}}}{\left(r_{iu}-{\hat{r}}_{iu}\right)}^{2}},$$

where $D_{\mathrm{test}}$ is the data of the test set.

**Figure 9:**
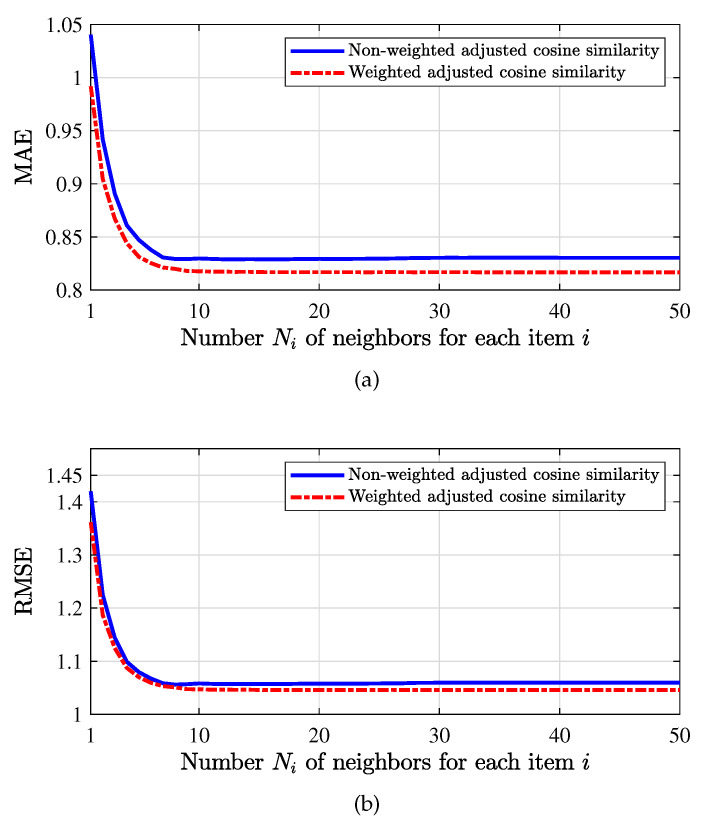
When the training set ratio is β=0.8, for non-weighted cosine similarity and weighted cosine similarity, compare the value of MAE and RMSE with different number $\left|N_{i}\right|$ of neighbors for each item, in which (**a**) is MAE and (**b**) is RMSE.

We set the training set ratio to be β=0.8. That is, 80 percent of data are used as the training set and 20 percent of data are used as the test data. We evaluate the prediction performance of both the traditional similarity and the proposed weighted similarity (cf. (43) and (44)) on the ML100k dataset in Figure 9.

In Figure 9a, we observe that the MAE under the weighted similarity is always smaller than that of the traditional similarity, which demonstrates that the proposed weighted similarity is more accurate and reasonable. As the number $\left|N_{i}\right|$ of neighbors of each item is increased, the MAE would decrease gradually and tend to some constant as the number of neighbors reaches about 10. In Figure 9b, similar conclusions can be obtained from the result on RMSE, which also shows the superiority of the proposed weighted similarity.

Since the proposed CUIS based recommendations are developed under the framework of collaborative filtering, it is necessary to mine the users’ preferences by capturing their interaction behaviors with items. At the time a user or an item first enters the system, there is no behavior or no purchase (i.e., cold start problems) recordings available, the system cannot explore users’ preferences through sufficient log information, and thus the recommendation quality would be poor. To solve this problem, therefore, the method in this paper should be combined with other types of algorithms (such as content-based recommendation), i.e., using hybrid approaches to provide more accurate recommendations.

## 5. Conclusions

In this paper, we try to improve the accuracy of recommendations by exploiting the difference among items and users. Based on the weighted similarity measure, we proposed a CUIS algorithm and the corresponding recommenders, which are shown to be effective. We mathematically proved that CUIS converges to the optimal solution. We also showed that recommenders based on the weighted similarity outperform recommenders using traditional non-weighted similarity. In our future work, we shall further investigate how a user likes items (how an item is liked by users) differently and design efficient schemes to calculate the corresponding weighting coefficients. Moreover, considering users’ drifting interests or user multi-interest modeling are also of interest.

## 6. Patents

Part of this work has been approved by Chinese invention patent “An efficient searching method for core-users and core-items in large-scale on-line merchandising”, with the authorization announcement number CN 113204713 B. The patent has also been submitted as an international PCT patent, with the international application number PCT/CN2021/143476. 

## Figures and Tables

**Table 1:** An example of rating matrix.

	Joe	John	Ali	Mike	Bob
Star Wars	5	5	5	*	3
WALL·E	4	*	5	5	3
Inception	4	3	5	*	2
The Lion King	*	3	*	4	*
Frozen	2	3	2	4	5

**Table 2:** Summary of notations.

$R\in R^{M\times N}$	Rating matrix
$\eta \in R^{M\times N}$	Importance degree matrix of items to users
$\rho \in R^{M\times N}$	Importance degree matrix of users to items
$U_{i}$	Set of users who like item *i*
$I_{u}$	Set of items liked by user *u*
⊙(u)	Core item of user *u*
⊙(i)	Core user of item *i*
$t_{uv}$	Traditional non-weighted user similarity of users *u* and *v*
$s_{ij}$	Traditional non-weighted item similarity of items *i* and *j*
${\hat{t}}_{uv}$	Weighted similarity of users *u* and *v*
${\hat{s}}_{ij}$	Weighted similarity of items *i* and *j*
$S_{u}$	Core user selection scheme
$S_{i}$	Core item selection scheme
${\hat{T}}_{u\to i}$	Total weighted user similarity centered at *u* for item *i*
${\hat{S}}_{i\to u}$	Total weighted item similarity centered at *i* for user *u*
${\hat{\hat{T}}}_{i}$	Total weighted user similarity centered at ⊙(i) for item *i*
${\hat{\hat{S}}}_{u}$	Total weighted item similarity centered at ⊙(u) for user *u*
*l*	Iteration rounds
${\hat{\hat{T}}}^{\left(l\right)}\in R^{M}$	The *l*-th each item-revenue
${\hat{\hat{S}}}^{\left(l\right)}\in R^{N}$	The *l*-th each user-revenue
$d^{\left(l\right)}\in R^{N+M}$	The *l*-th system-revenue
$V\left(\eta^{\left(0\right)}\right)\in R^{N+M}$	The total revenue of the system
*k*	Number of clusters
*K*	Number of recommended users or items
*F*	Number of users or items neighbors

**Table 3:** Weighting Matrices.

$\eta_{i\to u}$						$\rho_{u\to i}$					
	$u_{1}$	$u_{2}$	$u_{3}$	$u_{4}$	$u_{5}$		$u_{1}$	$u_{2}$	$u_{3}$	$u_{4}$	$u_{5}$
$i_{1}$	0.269	1	0.269	0	0	$i_{1}$	0.255	0.491	0.255	0	0
$i_{2}$	0.355	0	0.355	0.176	0	$i_{2}$	0.445	0	0.445	0.110	0
$i_{3}$	0.376	0	0.376	0	0	$i_{3}$	0.5	0	0.5	0	0
$i_{4}$	0	0	0	0.518	0	$i_{4}$	0	0	0	1	0
$i_{5}$	0	0	0	0.316	1	$i_{5}$	0	0	0	0.358	0.642

**Table 4:** Core Users and Core Items.

	$u_{1}$	$u_{2}$	$u_{3}$	$u_{4}$	$u_{5}$
⊙(u)	$i_{3}$	$i_{1}$	$i_{3}$	$i_{4}$	$i_{5}$
${\hat{\hat{S}}}_{u}$	7.971	8.769	7.971	5.789	3.917
	$i_{1}$	$i_{2}$	$i_{3}$	$i_{4}$	$i_{5}$
⊙(i)	$u_{2}$	$u_{1}$	$u_{1}$	$u_{4}$	$u_{5}$
${\hat{\hat{T}}}_{i}$	6.109	5.451	4	1	3.113

**Table 5:** Computational Complexity of CUIS and the Proposed Recommenders.

CUIS		$O({\mathrm{nnz}}^{2})$	
**Recommenders**			
CU-Rec	$O(\bar{d_{u}}m)$	CI-Rec	$O(\bar{d_{i}}n)$
CU-*k*MCL-Rec	$O(nkl_{k}+\bar{d_{\hat{u}}}m)$	CI-*k*MCL-Rec	$O(mkl_{k}+\bar{d_{\hat{i}}}n)$
WUS-PAF-Rec	$O(\bar{d_{i}}m)$	WIS-PAF-Rec	$O(\bar{d_{u}}n)$

**Table 6:** Statistics of the Two Datasets.

Dataset	#Ratings	#Items	#Users	Density
ML100k	100,000	1628	943	6.3%
ML1m	1,000,209	3706	6040	4.5%

**Table 7:** Confusion Matrix.

Ground Truth	Prediction Results	
	Recommended	Not Recommended
Relevant	True Positive (TP)	False Negative (FN)
Irrelevant	False Positive (FP)	True Negative (TN)

**Table 8:** Comparison of the Recommendation Performance of Using Different Weighting Scheme to Recommend 5 Users or Items in the Proposed Recommenders on ML100k.

Recommenders		CU-Rec	CI-Rec	WUS-PAF-Rec	WIS-PAF-Rec
Precision@5	NO	0.0207	0.0011	0.1398	0.0934
	VW	0.0143	0.0088	0.1769	0.1345
	IUF	0.0224	0.0176	0.2242	0.1565
	CUIS	**0.0231**	**0.0187**	**0.2278**	**0.1570**
Recall@5	NO	0.0069	0.0028	0.0260	0.0925
	VW	0.0070	0.0020	0.0368	0.1104
	IUF	0.0069	0.0035	0.0737	0.1446
	CUIS	**0.0101**	**0.0040**	**0.0764**	**0.1475**
NDCG@5	NO	0.0197	0.0097	0.1426	0.1109
	VW	0.0135	0.0074	0.1863	0.1477
	IUF	0.0223	0.0167	0.2418	0.1739
	CUIS	**0.0223**	**0.0190**	**0.2439**	**0.1742**
MRR@5	NO	0.0406	0.0019	0.2255	0.2409
	VW	0.0388	0.0079	0.3041	0.2864
	IUF	0.0458	0.0343	0.4219	0.3447
	CUIS	**0.0460**	**0.0421**	**0.4245**	**0.3514**

**Table 9:** Comparison of the Recommendation Performance of Using Different Weighting Scheme to Recommend 20 Users or Items in the Proposed Recommenders on ML100k.

Recommenders		CU-Rec	CI-Rec	WUS-PAF-Rec	WIS-PAF-Rec
Precision@5	NO	0.0279	0.0073	0.1590	0.0598
	VW	0.0263	0.0066	0.1378	0.0971
	IUF	0.0272	0.0239	0.1565	0.1053
	CUIS	**0.0282**	**0.0247**	**0.1609**	**0.1080**
Recall@5	NO	0.0337	0.0109	0.1924	0.1283
	VW	0.0258	0.0178	0.1114	0.1864
	IUF	0.0328	0.0200	0.1842	0.2376
	CUIS	**0.0339**	**0.0217**	**0.1959**	**0.2554**
NDCG@5	NO	0.0264	0.0055	0.1823	0.0750
	VW	0.0254	0.0042	0.1515	0.1117
	IUF	0.0258	0.0222	0.1814	0.1256
	CUIS	**0.0265**	**0.0235**	**0.1840**	**0.1272**
MRR@5	NO	0.0642	0.0089	0.4440	0.2541
	VW	0.0604	0.0081	0.3247	0.3049
	IUF	0.0639	0.0584	0.4463	0.3721
	CUIS	**0.0647**	**0.0661**	**0.4471**	**0.3759**

## Data Availability

Not applicable.
